# Supplementary material for: In vivo cisplatin-resistant neuroblastoma metastatic model reveals tumour necrosis factor receptor superfamily member 4 (TNFRSF4) as an independent prognostic factor of survival in neuroblastoma
Source: PLoS One. 2024 May 29;19(5):e0303643. doi: 10.1371/journal.pone.0303643 (PMC11135766; doi:10.1371/journal.pone.0303643)
Supplement: S1 Fig — (PDF) [file pone.0303643.s001.pdf]

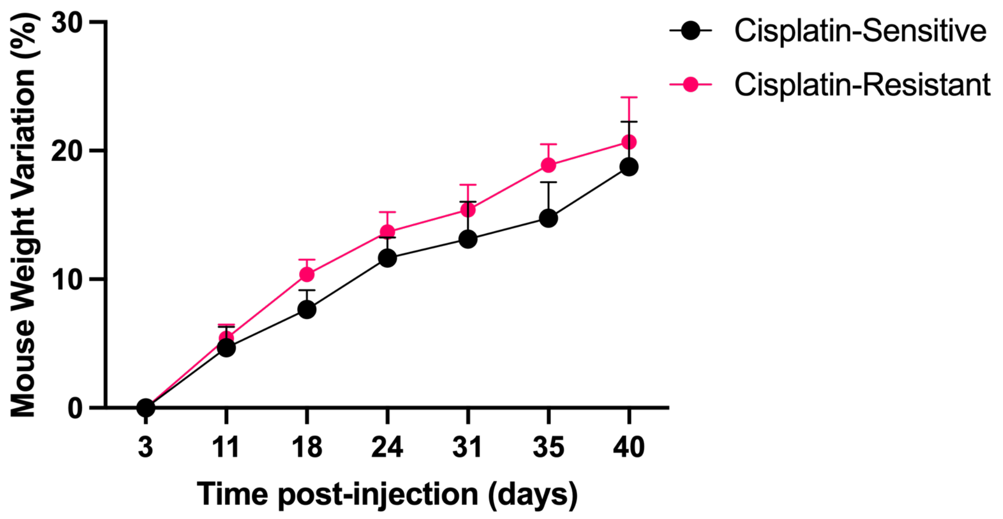

Fig S1. Mouse weight variation in the murine xenograft model. Mice were routinely weighed postinjection, and the mouse weight variation (%) was calculated. No significant differences in mouse weight were detected between the two groups.
(*p ≤ 0.05; **p ≤ 0.01; ***p≤ 0.001; ****p ≤ 0.0001).
