# Supplementary material for: In vivo cisplatin-resistant neuroblastoma metastatic model reveals tumour necrosis factor receptor superfamily member 4 (TNFRSF4) as an independent prognostic factor of survival in neuroblastoma
Source: PLoS One. 2024 May 29;19(5):e0303643. doi: 10.1371/journal.pone.0303643 (PMC11135766; doi:10.1371/journal.pone.0303643)
Supplement: S2 Fig — (PDF) [file pone.0303643.s002.pdf]

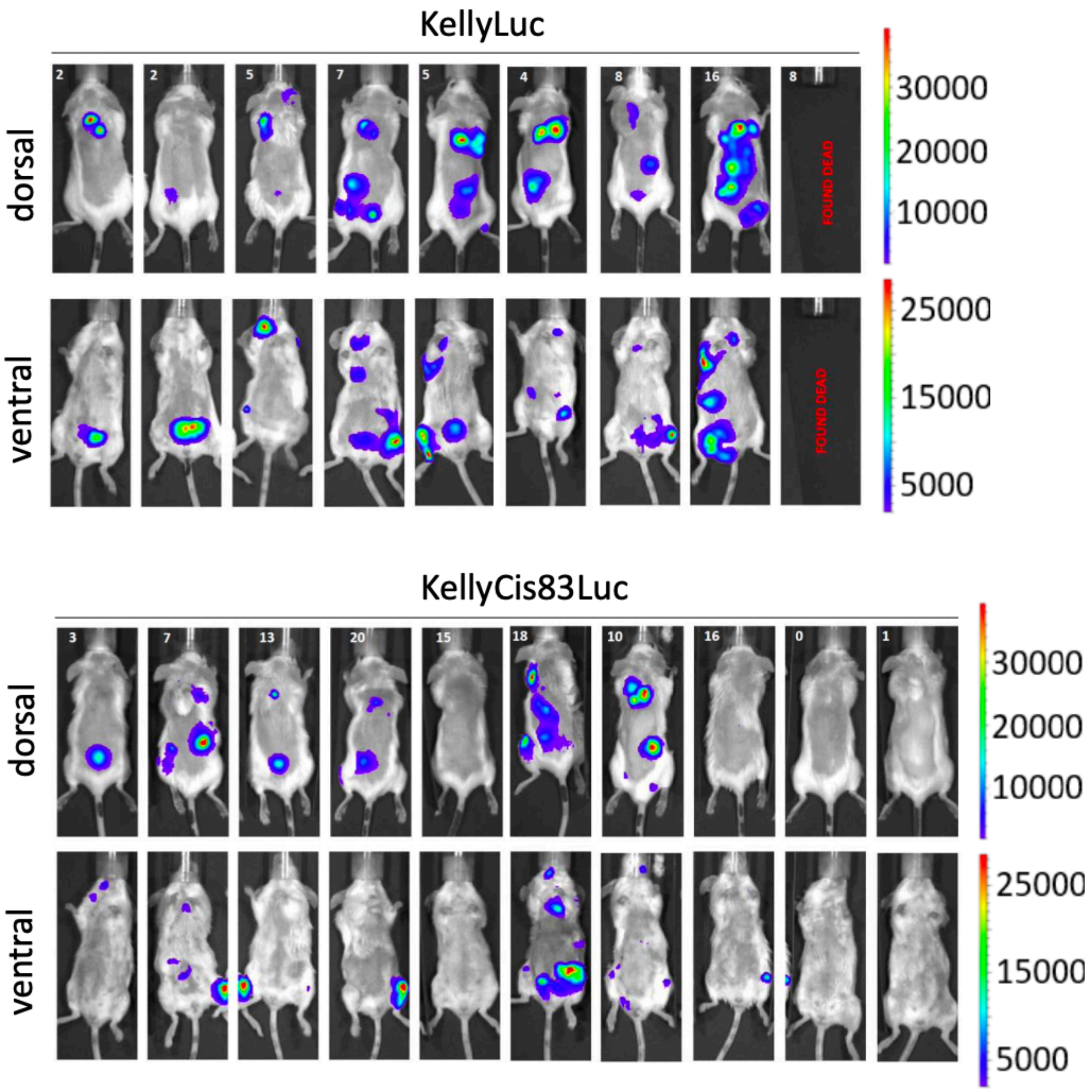

Fig S2. Cisplatin-resistant KellyCis83Luc tail-vein-injected xenografts have more metastatic foci than drug-sensitive KellyLuc xenografts. IVIS imaging of the remaining four KellyLuc-injected mice and six KellyCis83Luc-injected mice showed that luciferase-containing metastases were used to determine the appropriate time to sacrifice. The total number of metastases per mouse was confirmed by necropsy in the top left corner. Luciferase scale - dorsal min: 1992, max: 28575; ventral min:1992, max: 39398
