# Supplementary material for: In vivo cisplatin-resistant neuroblastoma metastatic model reveals tumour necrosis factor receptor superfamily member 4 (TNFRSF4) as an independent prognostic factor of survival in neuroblastoma
Source: PLoS One. 2024 May 29;19(5):e0303643. doi: 10.1371/journal.pone.0303643 (PMC11135766; doi:10.1371/journal.pone.0303643)
Supplement: S3 Fig — (PDF) [file pone.0303643.s003.pdf]

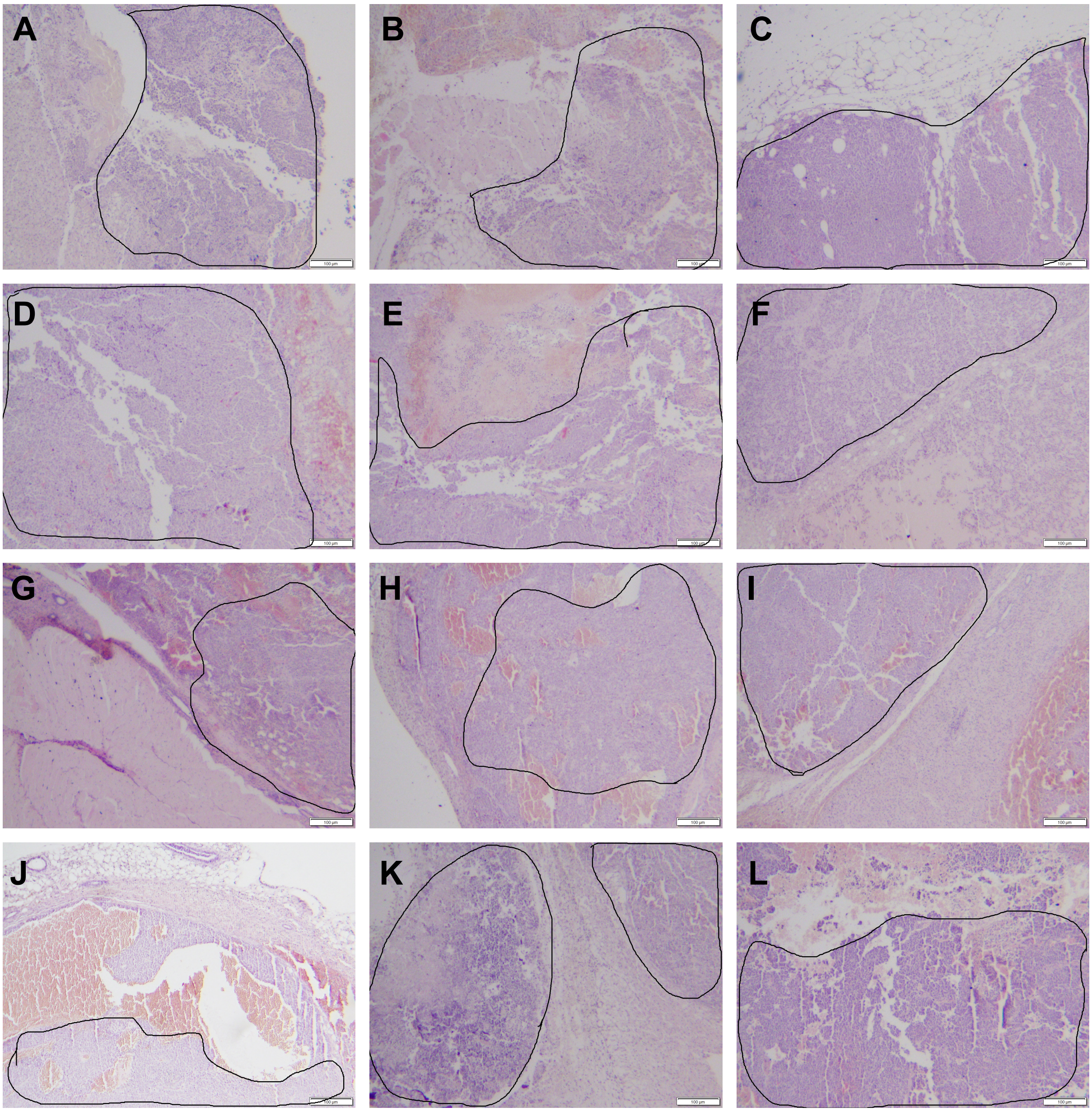

Fig S3. Haematoxylin and eosin staining of tumours resected from the drug-resistant neuroblastoma xenograft model. FFPE sections of tumours resected from mice injected with KellyLuc (A-F) and KellyCis83Luc (G-L) cells were stained with haematoxylin and eosin (H&E) to identify tumour cell-enriched regions (outlined). These tumour cell-enriched regions were then macrodissected for RNA sequencing.
