# Supplementary material for: In vivo cisplatin-resistant neuroblastoma metastatic model reveals tumour necrosis factor receptor superfamily member 4 (TNFRSF4) as an independent prognostic factor of survival in neuroblastoma
Source: PLoS One. 2024 May 29;19(5):e0303643. doi: 10.1371/journal.pone.0303643 (PMC11135766; doi:10.1371/journal.pone.0303643)
Supplement: S4 Fig — (PDF) [file pone.0303643.s004.pdf]

A

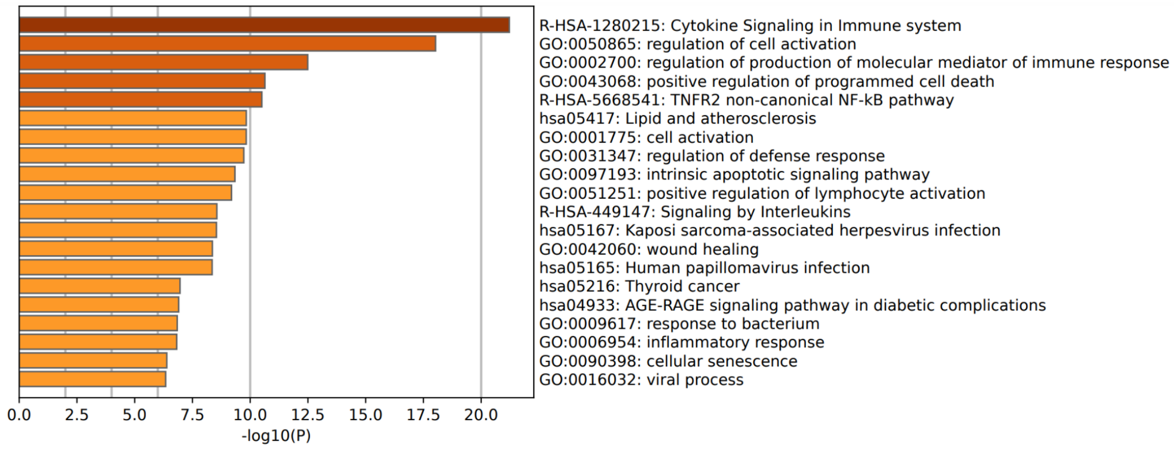

B

Cytokine Signalling in Immune System (N=19)
TNFRSF8, CDKN1A, FN1, HLA-A, HMOX1, HRAS, IL11, IRAK2, IRF7, LTBR, PRKCD, RAG1, CCL2, TNFRSF1B, TRAF2, TNFRSF4, TNFRSF14, TNFRSF11A, SOCS3

Regulation of Cell Activation (N=19)
ADORA2A, RUNX3, CDKN1A, CD55, FN1, HLA-A, HMOX1, PRKCD, RAG1, CCL2, TCF7, TNFRSF1B, TNFRSF4, TNFRSF14, MERTK, SPINK5, TICAM1, PPARG ITGA5

Regulation of Production of Molecular Mediator of Immune Response (N=17)
CD55, HLA-A, HMOX1, TNFRSF1B, TRAF2, TNFRSF4, TNFRSF14, SPINK5, TICAM1, TNFRSF8, HRAS, IRF7, POU2F2, PPARG, PAX5, CCL2, GNLY

Fig S4. Metascape Pathway and Process Enrichment Analysis. A) Terms with a *p* value <0.01, a minimum count of 3, and an enrichment factor >1.5 are collected and grouped into clusters based on their membership similarities. *p* values are converted to log base 10, and processes with the highest - log10(P) are graphed on the bar chart. The most statistically significant term within a cluster is chosen to represent the cluster. B) Candidate genes present in the top three enriched processes.
