# Supplementary material for: In vivo cisplatin-resistant neuroblastoma metastatic model reveals tumour necrosis factor receptor superfamily member 4 (TNFRSF4) as an independent prognostic factor of survival in neuroblastoma
Source: PLoS One. 2024 May 29;19(5):e0303643. doi: 10.1371/journal.pone.0303643 (PMC11135766; doi:10.1371/journal.pone.0303643)
Supplement: S5 Fig — (PDF) [file pone.0303643.s005.pdf]

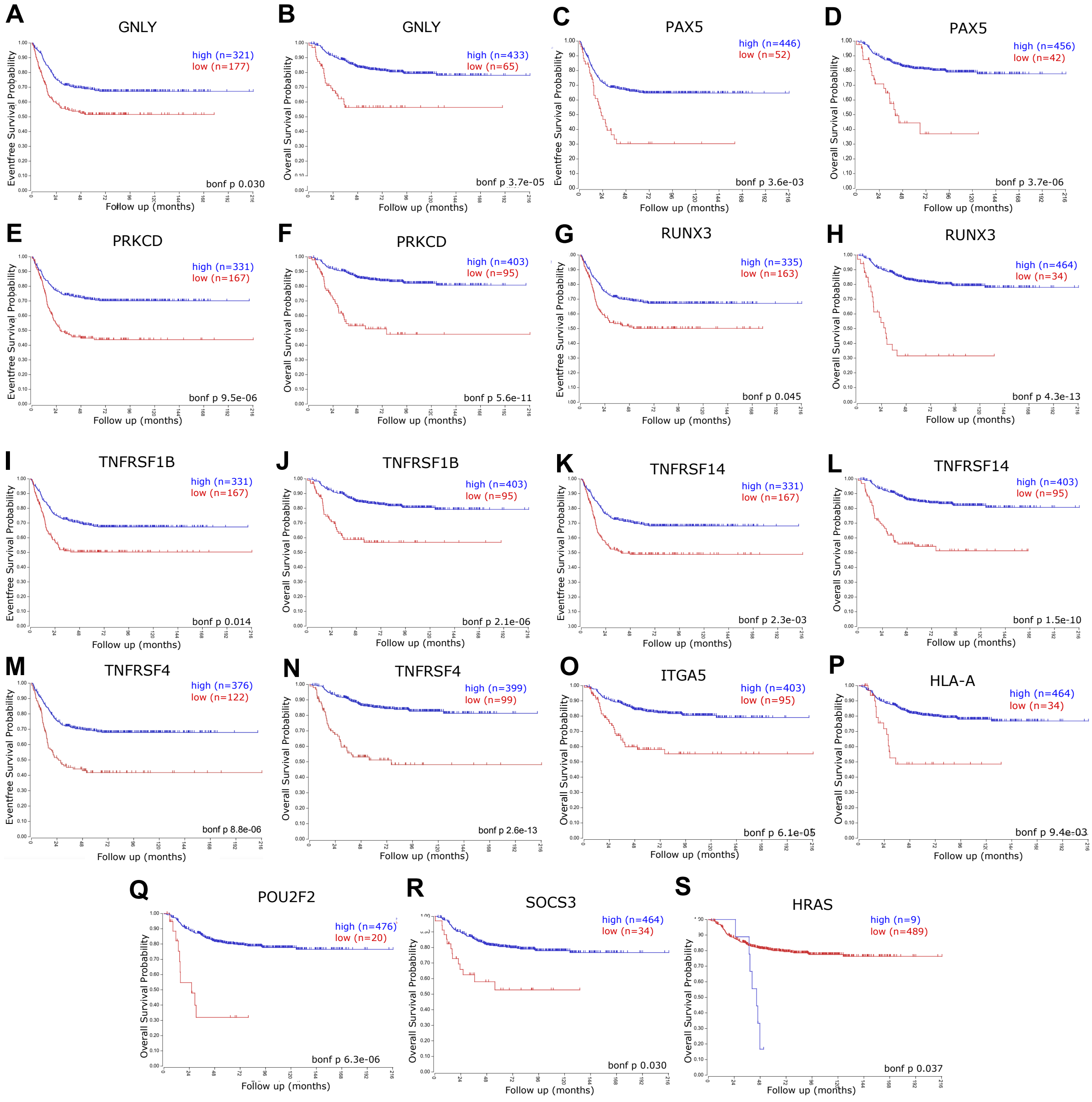

Fig S5. Kaplan–Meier survival curves for shortlisted genes that demonstrated clinical significance. The association between the candidate genes and EFS and OS was assessed in the SEQC cohort of 498 neuroblastomas in R2GAVP(22). Expression was split into "high" and "low" groups via the Scan function in R2GAVP. In addition to *TNFRSF4* (M-N), six other genes were clinically significant for both EFS and OS: *GNLY, PAX5, PRKCD, RUNX3, TNFRSF1B* and *TNFRSF14* (A-L). For all of these genes, lower expression significantly reduced the survival probabilities for both EFS and OS (Bonferroni-adjusted $p \leq 0.05$). There were also 5 genes that were significant for OS but not EFS: *ITGA5, HLA-A, POU2F2, SOCS3* and *HRAS* (O-S). Low expression of *ITGA5, HLA-A, POU2F2* and *SOCS3* significantly reduced OS probabilities, while high expression of *HRAS* significantly reduced OS probabilities (Bonferroni-adjusted $p \leq 0.05$).
