# Supplementary material for: In vivo cisplatin-resistant neuroblastoma metastatic model reveals tumour necrosis factor receptor superfamily member 4 (TNFRSF4) as an independent prognostic factor of survival in neuroblastoma
Source: PLoS One. 2024 May 29;19(5):e0303643. doi: 10.1371/journal.pone.0303643 (PMC11135766; doi:10.1371/journal.pone.0303643)
Supplement: S6 Fig — (PDF) [file pone.0303643.s006.pdf]

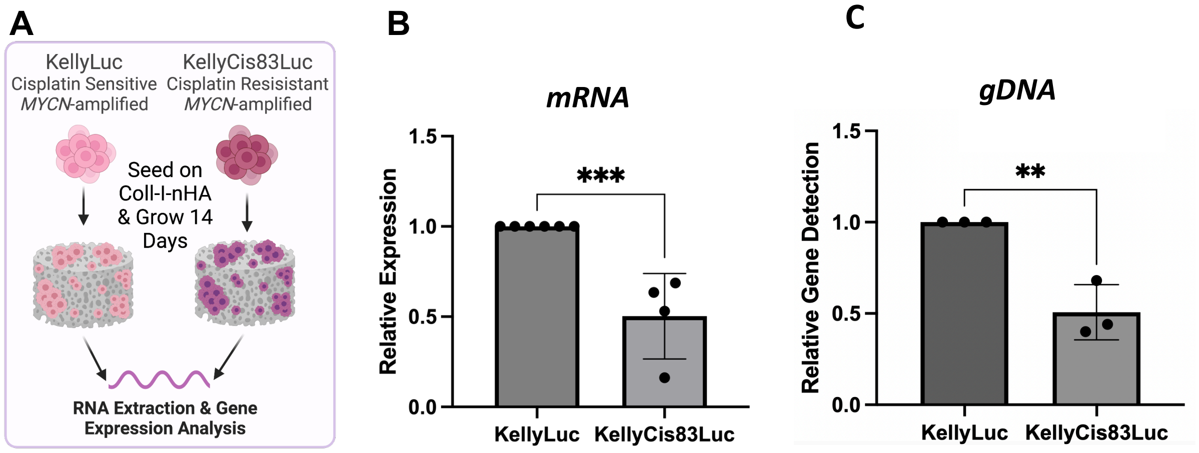

Fig S6: ***RT-qPCR validation of expression trends in KellyLuc and KellyCis83Luc cells grown in 3D in vitro model*.** A) A collagen-based scaffold model was used to grow KellyLuc and KellyCis83Luc cells in a 3D *in vitro* microenvironment for 14 days. RNA was extracted from cells grown on scaffolds for validation of *TNFRSF4* expression trends via RT-qPCR. B) *TNFRSF4* was significantly downregulated (p=0.0007) in KellyCis83Luc cells grown on collagen-based scaffolds compared to KellyLuc cells. C) *TNFRSF4* was significantly downregulated (p=0.0049) in KellyCis83Luc genomic DNA cells compared to KellyLuc genomic DNA. Image created with BioRender.com.
