# Supplementary material for: In vivo cisplatin-resistant neuroblastoma metastatic model reveals tumour necrosis factor receptor superfamily member 4 (TNFRSF4) as an independent prognostic factor of survival in neuroblastoma
Source: PLoS One. 2024 May 29;19(5):e0303643. doi: 10.1371/journal.pone.0303643 (PMC11135766; doi:10.1371/journal.pone.0303643)
Supplement: S7 Fig — Correlation between MYCN and TNFRSF4 expression generated in R2GAVP (22) using the Westermann cohort of 579 neuroblastomas (A) and the Fischer cohort of 223 neuroblastoma (B). (PDF) [file pone.0303643.s007.pdf]

A

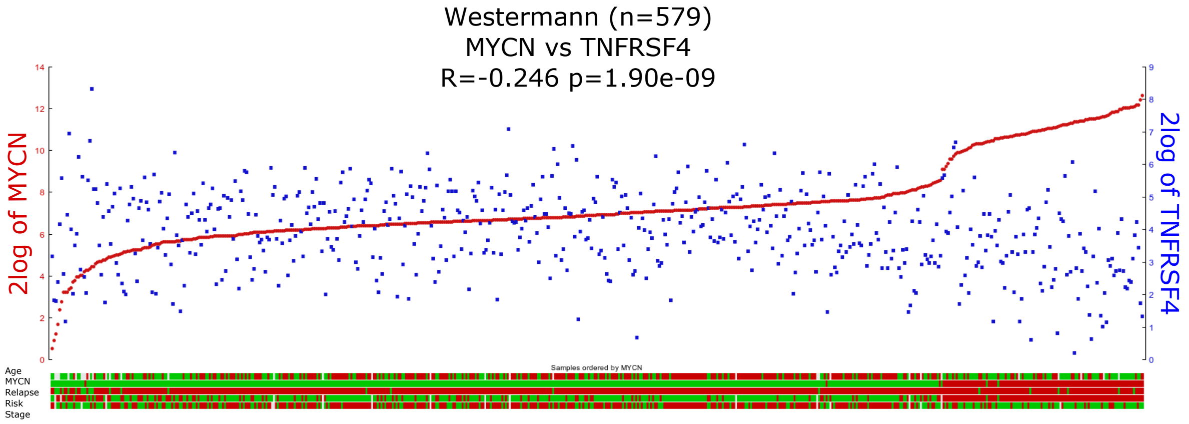

B

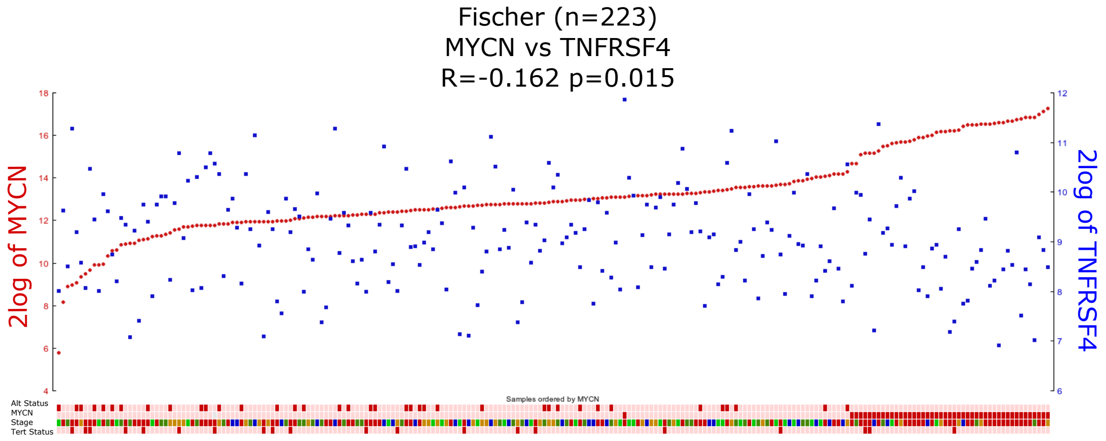

Fig S7. Correlation between *MYCN* and *TNFRSF4* expression generated in R2GAVP (22) using the Westermann cohort of 579 neuroblastomas (A) and the Fischer cohort of 223 neuroblastoma (B).
