# Supplementary material for: In vivo cisplatin-resistant neuroblastoma metastatic model reveals tumour necrosis factor receptor superfamily member 4 (TNFRSF4) as an independent prognostic factor of survival in neuroblastoma
Source: PLoS One. 2024 May 29;19(5):e0303643. doi: 10.1371/journal.pone.0303643 (PMC11135766; doi:10.1371/journal.pone.0303643)
Supplement: S8 Fig — (PDF) [file pone.0303643.s008.pdf]

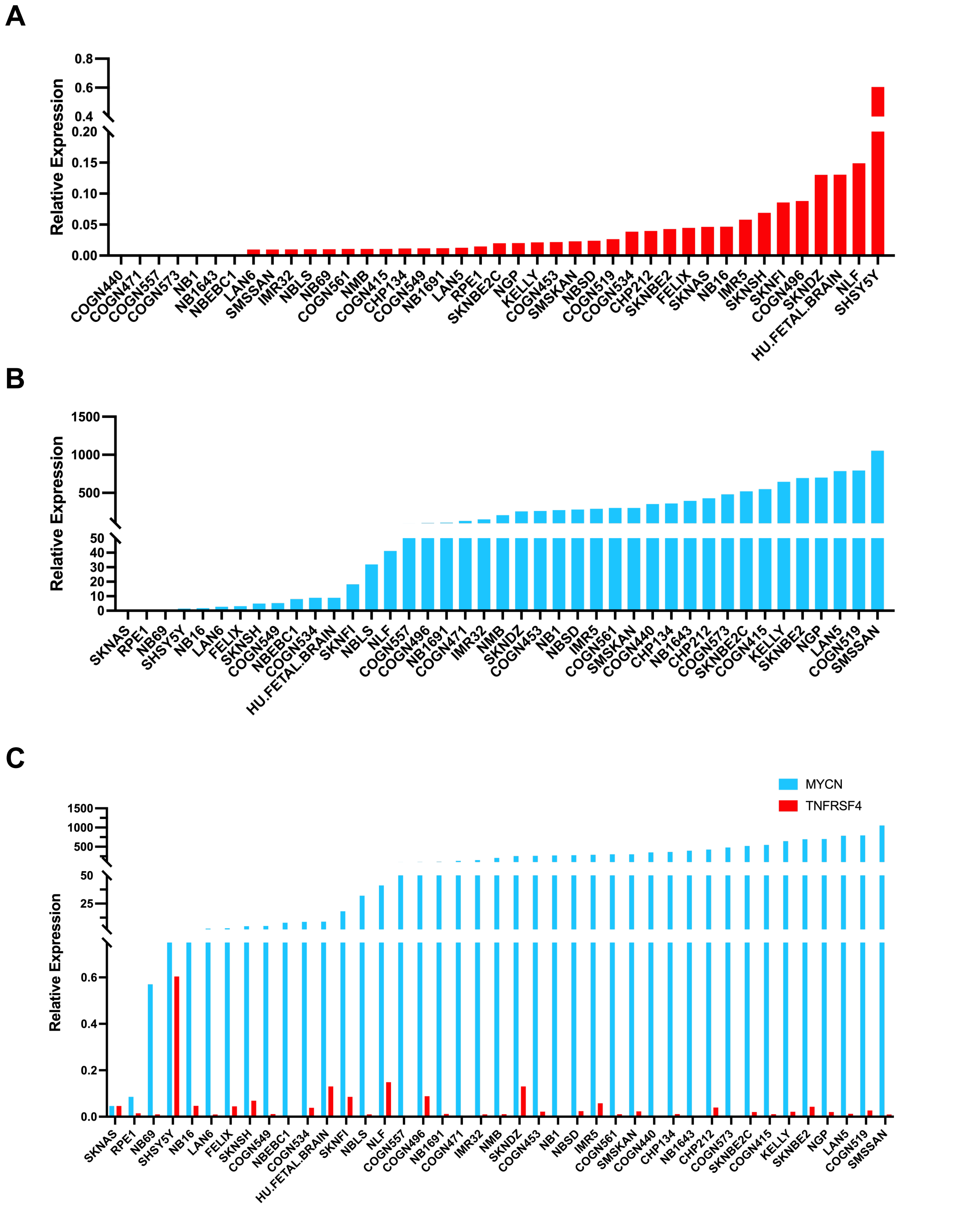

Fig S8. Expression of *TNFRSF4* and *MYCN* in a panel of neuroblastoma cell lines. A study by Harenza et al 2017 carried out whole transcriptomic profiling on a panel of commonly used neuroblastoma cell lines. A) *TNFRSF4* expression was overall low in the neuroblastoma cell lines, with the highest expression in SHSY5Y cells, almost 29-fold higher than that in Kelly cells. B) *MYCN* expression was overall higher in the neuroblastoma cell lines. Fourteen cell lines were not *MYCN*-amplified and displayed lower expression, while 27 cell lines were *MYCN*-amplified (left to right) with the highest expression in SMSSAN cells. C) When both *TNFRSF4* and *MYCN* are plotted on the same graph, with cell lines arranged in order of *MYCN* expression, there is no obvious trend between *MYCN* and *TNFRSF4* expression levels. Original figures from data by Harenza et al.[DOI: 10.1038/sdata.2017.33].
