# Supplementary material for: In vivo cisplatin-resistant neuroblastoma metastatic model reveals tumour necrosis factor receptor superfamily member 4 (TNFRSF4) as an independent prognostic factor of survival in neuroblastoma
Source: PLoS One. 2024 May 29;19(5):e0303643. doi: 10.1371/journal.pone.0303643 (PMC11135766; doi:10.1371/journal.pone.0303643)
Supplement: S9 Fig — (PDF) [file pone.0303643.s009.pdf]

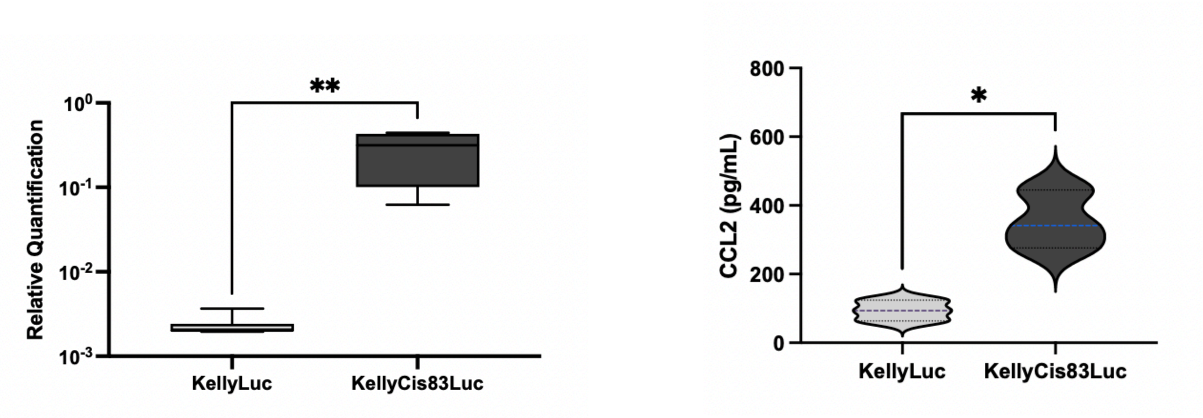

Fig S9. Expression of CCL2 in a cisplatin-resistant metastatic model of neuroblastoma. (A) Validation of *CCL2* expression in fresh-frozen KellyLuc and KellyCis83Luc xenografts by RT–qPCR. (B) CCL2 secretion by KellyLuc and KellyCis83Luc cells grown *in vitro* quantified by ELISA. Two-tailed unpaired T tests were performed in GraphPad Prism to detect significant differences in expression (*$p \leq 0.05$; **$p \leq 0.01$; ***$p \leq 0.001$; ****$p \leq 0.0001$).
