# Supplementary material for: In vivo cisplatin-resistant neuroblastoma metastatic model reveals tumour necrosis factor receptor superfamily member 4 (TNFRSF4) as an independent prognostic factor of survival in neuroblastoma
Source: PLoS One. 2024 May 29;19(5):e0303643. doi: 10.1371/journal.pone.0303643 (PMC11135766; doi:10.1371/journal.pone.0303643)
Supplement: S1 Table — (PDF) [file pone.0303643.s010.pdf]

Table S1: Available identifiers for R2 tumour neuroblastoma cohorts.

Annotation	SEQC - custom - ag44kcwolf (N=498) [GSE62564]	Fischer - custom - ag44kcwolf (N=223) [GSE120572]
**Sex**	Male (n=287)<br><br>Female (n=211)	
**Age at Diagnosis (months)**	<18 (n=300)<br><br>≥18 (n=198)	
***MYCN* Status**	MNA (n=92)<br><br>Non-MNA (n=401)<br><br>No Info. (n=5)	MNA (n=46)<br><br>Non-MNA (n=176)<br><br>No Info. (n=1)
**COG Risk Group**	High-risk (n=176)<br><br>Low-risk (n=322)	
**INSS Stage**	Stage 1 (n=121)<br><br>Stage 2 (n=78)<br><br>Stage 3 (n=63)<br><br>Stage 4 (n=183)<br><br>Stage 4S (n=53)	Stage 1 (n=29)<br><br>Stage 2 (n=39)<br><br>Stage 3 (n=36)<br><br>Stage 4 (n=89)<br><br>Stage 4S (n=30)
**Disease Course**	Favourable (n=181)<br><br>Unfavourable (n=91)<br><br>No Info. (n=226)	
**Death from Disease**	Yes (n=105)<br><br>No (n=393)	
**Tumour Progression**	Yes (n=183)<br><br>No (n=315)	
***ALT* Status**		Positive (n=38)<br><br>Negative (n=185)
**Chromosome 1p Deletion**		Deleted (n=67)<br><br>Normal (n=137)<br><br>No Info. (n=19)
**Chromosome 11q Deletion**		Deleted (n=46)<br><br>Normal (n=18)<br><br>No Info. (n=159)

When assessing the clinical relevance of candidate genes, the neuroblastoma cohorts SEQC (N=498) and Fischer (N=223) were referenced in R2, with each cohort offering different cohort annotations and subgroups. Unfavourable disease course was defined as follows: patient died despite intensive chemotherapy; favourable disease course was defined as patient survived without chemotherapy for at least 1000 days postdiagnosis. MNA = *MYCN*-amplified.
