# Supplementary material for: In vivo cisplatin-resistant neuroblastoma metastatic model reveals tumour necrosis factor receptor superfamily member 4 (TNFRSF4) as an independent prognostic factor of survival in neuroblastoma
Source: PLoS One. 2024 May 29;19(5):e0303643. doi: 10.1371/journal.pone.0303643 (PMC11135766; doi:10.1371/journal.pone.0303643)
Supplement: Attachment — Submitted filename: OPiskareva Response to Reviewers_090424.pdf [file pone.0303643.s011.pdf]

Dear Editor and Reviewers,

On behalf of all co-author, I would like to thank the reviewers for their time and expertise in evaluating our manuscript. I appreciate the comprehensive and constructive appraisal of our work and positive comments.

I hope that Reviewer's concerns have been addressed and added more clarity to the submitted manuscript.

All our responses are in red below. A corresponding reference to the manuscript page is provided. All changes in the manuscript body are tracked.

Yours sincerely,

Olga Piskareva

Corresponding author.

Journal Requirements:

When submitting your revision, we need you to address these additional requirements.

1. Please ensure that your manuscript meets PLOS ONE's style requirements, including those for file naming. The PLOS ONE style templates can be found at

https://journals.plos.org/plosone/s/file?id=wjVg/PLOSOne_formatting_sample_main_body.pdf and
https://journals.plos.org/plosone/s/file?id=ba62/PLOSOne_formatting_sample_title_authors_affiliations.pdf

We have checked this information and revised the manuscript accordingly.

2. To comply with PLOS ONE submissions requirements, in your Methods section, please provide additional information regarding the experiments involving animals and ensure you have included details on (1) methods of sacrifice, (2) methods of anesthesia and/or analgesia, and (3) efforts to alleviate suffering.

We have checked this information.

3. We note that the grant information you provided in the 'Funding Information' and 'Financial Disclosure' sections do not match.

When you resubmit, please ensure that you provide the correct grant numbers for the awards you received for your study in the 'Funding Information' section.

We have checked this information and revised the manuscript accordingly.

4. Thank you for stating the following financial disclosure:

"O.P. received support for this project through Neuroblastoma UK, RCSI Strategic Academic Recruitment (StAR) Programme, Health Research Board - The Conor Foley Neuroblastoma Cancer Research Foundation (HRCI-HRB-2022-013). C.G. was funded by Irish Research Council Postgraduate Programme (GOIPG/2019/3220), R.S. - Irish Research Council - The Conor Foley Neuroblastoma Cancer Research Foundation (EPSPG/2021/95). M.S. was funded by Instituto de Salud Carlos III through the projects "ICI21/00076", "PI23/01144" and "PMP21/00073" (Co-funded by the European Regional Development Fund/European Social Fund; "A way to make Europe"/ "Investing in your future"). L.D.-J. is recipient of a Ramón y Cajal scheme (Grant No. RyC-2021-034346-I), funded by the Spanish Ministry for Science and Innovation (MCIN)"

Please state what role the funders took in the study. If the funders had no role, please state: ""The funders had no role in study design, data collection and analysis, decision to publish, or preparation of the manuscript."" If this statement is not correct you must amend it as needed. We have added this statement and updated the grant numbers, p28.

Please include this amended Role of Funder statement in your cover letter; we will change the online submission form on your behalf.

5. Please be informed that funding information should not appear in the Acknowledgments section or other areas of your manuscript. We will only publish funding information present in the Funding Statement section of the online submission form. Please remove any funding-related text from the manuscript.

We have checked the manuscript and confirmed that funding information appears in the Funding Statement section.

6. When completing the data availability statement of the submission form, you indicated that you will make your data available on acceptance. We strongly recommend all authors decide on a data sharing plan before acceptance, as the process can be lengthy and hold up publication timelines. Please note that, though access restrictions are acceptable now, your entire data will need to be made freely accessible if your manuscript is accepted for publication. This policy applies to all data except where public deposition would breach compliance with the protocol approved by your research ethics board. If you are unable to adhere to our open data policy, please kindly revise your statement to explain your reasoning and we will seek the editor's input on an exemption. Please be assured that, once you have provided your new statement, the assessment of your exemption will not hold up the peer review process.

We made the HTG EdgeSeq immuno-oncology panel mRNA profiling data available at R2: Genomics Analysis and Visualization Platform. P 28.

7. Your ethics statement should only appear in the Methods section of your manuscript. If your ethics statement is written in any section besides the Methods, please delete it from any other section.

We removed the duplication from p 28. The ethics statement appears in the Methods section.

8. Please include captions for your Supporting Information files at the end of your manuscript, and update any in-text citations to match accordingly. Please see our Supporting Information guidelines for more information: http://journals.plos.org/plosone/s/supporting-information.

We have checked this information and revised the manuscript accordingly.

Additional Editor Comments:

Both reviewers agreed the manuscript to be interesting. However, they also raised concerns regarding the presentation, analysis, and interpretation of the data. They also noted a lack of data and discussion to fully support the authors' hypothesis and claims made in the manuscript. Additionally, the reviewers identified issues with some of the experimental designs, including the animal model. It is important to address each of these concerns meticulously and provide successful responses.

[Note: HTML markup is below. Please do not edit.]

Reviewers' comments:

Reviewer's Responses to Questions

Comments to the Author

1. Is the manuscript technically sound, and do the data support the conclusions?

The manuscript must describe a technically sound piece of scientific research with data that supports the conclusions. Experiments must have been conducted rigorously, with appropriate controls, replication, and sample sizes. The conclusions must be drawn appropriately based on the data presented.

Reviewer #1: Yes

Reviewer #2: Partly

---

2. Has the statistical analysis been performed appropriately and rigorously?

Reviewer #1: Yes

Reviewer #2: No

---

3. Have the authors made all data underlying the findings in their manuscript fully available?

The PLOS Data policy requires authors to make all data underlying the findings described in their manuscript fully available without restriction, with rare exception (please refer to the Data Availability Statement in the manuscript PDF file). The data should be provided as part of the manuscript or its supporting information, or deposited to a public repository. For example, in addition to summary statistics, the data points behind means, medians and variance measures should be available. If there are restrictions on publicly sharing data—e.g. participant privacy or use of data from a third party—those must be specified.

Reviewer #1: Yes

Reviewer #2: No

---

4. Is the manuscript presented in an intelligible fashion and written in standard English?

PLOS ONE does not copyedit accepted manuscripts, so the language in submitted articles must be clear, correct, and unambiguous. Any typographical or grammatical errors should be corrected at revision, so please note any specific errors here.

Reviewer #1: Yes

Reviewer #2: Yes

---

5. Review Comments to the Author

Please use the space provided to explain your answers to the questions above. You may also include additional comments for the author, including concerns about dual publication,

research ethics, or publication ethics. (Please upload your review as an attachment if it exceeds 20,000 characters)

Reviewer #1: This manuscript utilizes a murine model of neuroblastoma to compare a parental cell line versus its cisplatin resistant derivative to assess a possible impact on metastasis. The authors discover a disproportional metastatic tumor load associated with cisplatin resistance and identify dysregulated genes in these tumors associated with clinical relevance. The gene TNFRSF4 was shown to correlate with poor prognostic outcomes, though not risk of recurrence. Data indicated that TNFRSF4 did not appear to be regulated by changes in MYCN expression and the knockdown of TNFRSF4 expression did not significantly affect cell viability in vitro.

Here are my comments:

Major Comments:

1) On page 19, the authors indicate the following in regard to multivariate Cox regression analysis:
"The increased likelihood of recurrence based on Low expression of TNFRSF4 was no longer significant in this analysis, but this variable remained significant at increasing the likelihood of death 1.7-fold (p=0.007). Then, TNFRSF4 Low expression was an independent prognostic factor of survival in neuroblastoma."

If TNFRSF4 is no longer considered significant to the likelihood of recurrence, what role would you speculate TNFRSF4 is responsible for in the increased likelihood of death?

One would assume this role must be different from the roles of the remaining hazards, which all correlated with both "Risk of Recurrence" and "Risk of Death".

We have carefully considered the reviewer comment and provide our reasoning below.

Kaplan-Meier analysis evidenced that Low expression of TNFRSF4 significantly reduced survival probabilities in neuroblastoma, in the SEQC cohort of 498 neuroblastomas, in terms of both OS (*p=1.53e-09*) and EFS (*p=4.2e-04*) (Figure 4A-B of the manuscript).

As stated by the reviewer, once the relation between Low TNFRSF4 expression and patient outcome was evidenced, additionally we performed Cox univariate and multivariate analysis to determine if TNFRSF4 is an independent predictor of EFS and OS within the studied population.

In the univariate analysis, TNFRSF4 HR was statistically significant for both recurrence and death (Figure 5 of the manuscript).

However, the marker is a more powerful predictor of the death event (OS), as can be observed from the ROC curves for both OS and EFS:

For the OS, the is AUC= 0.657 (*p= 7.8 x10*$^{-7}$):

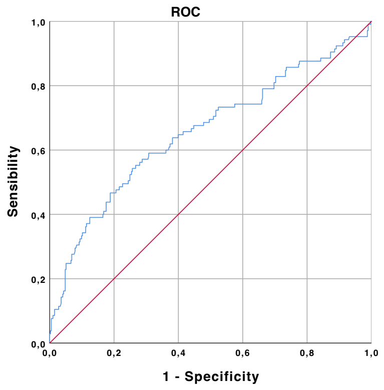

For the EFS, AUC= 0.564 (*p= 0.018*):

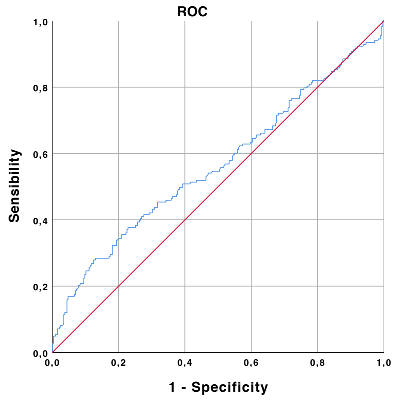

This is also why, the Low/High TNFRSF4 expression for the analysis was generated based on Youden Index for the OS event.

Cox regression analysis allows for a better understanding of the predictive value of the marker by comparing it to the basic clinical variables defined for the patient risk: MYCN amp/no-amp, age, and ISSN stage. As a survival marker (OS), it provides a specific term to the multivariate Cox constructed model, thus improving the survival prognosis made from the above mentioned three basic clinical variables. However, as a marker of event (EFS), its value is more limited since this global potential observed on a univariate basis is not maintained at a global level (*p=0.211*) when studied in a multivariate context that includes MYCN amp/no-amp.

In fact, the three bivariate Cox regression analysis with TNFRSF4 and the three clinical variables already mentioned have different behaviors: when including MYCN amp/no-amp, the model does not retain the TNFRSF4 variable although it is close to significance (*p=0.083*). TNFRSF4 is retained in the regression analysis model when including both the age (*p<0.001*) or the ISSN stage (*p<0.001*).
Tables illustrating these results can be shown as follows:

Bivariate regression analysis (TNFRSF4 & Age)

**Factors**	**Event free survival (recurrence)**	
	**HR (95% CI)**	***P* value**
TNFRSF4 (Low vs High)	1.439 (1.067-1.940)	0.017
Age (≥18 months vs <18 months)	2.899 (3.937-2.141)	0.000

Abbreviations: HR, hazard ratio

Bivariate regression analysis (TNFRSF4 & ISSN stage)

**Factors**	**Event free survival (recurrence)**	
	**HR (95% CI)**	***P* value**
TNFRSF4 (Low vs High)	1.423 (1.056-1.918)	0.021
ISSN Stage (4 vs others)	3.726 (2.746-5.056)	0.000

Abbreviations: HR, hazard ratio

Bivariate regression analysis (TNFRSF4 & MYCN amp no-amp)

**Factors**	**Event free survival (recurrence)**	
	**HR (95% CI)**	***P* value**
TNFRSF4 (Low vs High)	1.319 (0.964-1.806)	0.084
MYCN (amp vs no-amp)	2.941 (2.113-4.095)	0.000

Abbreviations: HR, hazard ratio

In this cohort, regarding the Cox multivariate regression model, MYCN amp/no-amp variable would be the cause why TNFRSF4 is not retained by the model. The two variables show interrelation in the study. However, more studies in larger cohorts, would be required to deep in the role of the gene of interest as a marker of tumour event in neuroblastoma.

2) In Figure 7 (Section 3.7, page 22), the authors indicated that siRNA knockdown of KIF11 in both Kelly and SK-N-AS neuroblastoma cells served as a positive control for cell viability and proliferation. Figure 7D corroborated this result. However, siRNA knockdown of TNFRSF4 (confirmed in Figure 7E) revealed minimal effects in the neuroblastomas. Unfortunately, that leaves the manuscript with no functional assay and therefore, no data to suggest what role TNFRSF4 plays in the predicted "increased likelihood of death" witnessed in patients. Can the authors offer any assay which helps point in the direction of a possible mechanism of action?

While the standard viability and proliferation assays were insufficient to conclude on the biological function of OX40 in neuroblastoma cells, they did provide a reference point to search for other potential roles. Other test that could link TNFRSF4 with aggressiveness could be changes in the interaction with immune cells. For example:

- Changes in the sensitivity to death ligands (FasL, TNFalpha)

- Changes in the activity of the NFkB pathway, that usually discriminates between pro-survival and pro-death signals.
- Changes in the released cytokine profile.

While we believe that this could be an interesting experiment it is rather out of scope of the present work. We apologise for not giving more insights in this matter and we added a sentence in the discussion to encourage future work to answer this question. P22

3) As a follow-up to Comment #2, given the volume of RNA Sequencing data you have acquired and the use of pathway analysis performed (using iDEP, for instance), could this data help to identify a relevant dysregulated pathway for a predicted function of TNFRSF4 in neuroblastoma mortality?

Unfortunately, the volume of acquired data is limited. We used a predefined panel of immune-oncology markers (549 genes and only 36 genes were significantly dysregulated). 36 genes is not sufficient to predict a relevant dysregulated pathway for a predicted function of TNFRSF4 in neuroblastoma mortality.

4) In the first paragraph of the Discussion (page 24), the authors indicate:
"Our study identified a potential relationship between the drug-resistant phenotype, which causes relapse in approximately 50% of neuroblastomas, the formation of a sentinel lymph node microenvironment during tumour metastasis and immune-related gene expression alterations".

I apologize, but can you clarify exactly what "immune-related gene expression alterations" were covered in this manuscript so far?

Thank you for this observation, which may be confusing. We revised this phrase throughout the text and replaced with *assessed immune-oncology genes*

Section 3.4 (page 15 – 16) indicates the title:
"Immune-Related Genes are Associated with Neuroblastoma Outcomes"

This section mentions "candidate genes" associated with "neuroblastoma aggressiveness" and assumes "a direct neuroblastoma – immune cell interaction would be facilitated by either proteins secreted or expressed on the cancer cell surface". However, the reader has no indication exactly how these candidates are immune related. Since this section deals with "Immune-Related Genes", shouldn't we know how they are immune related?

We agree that this section needs more wording clarity. We revised this section and referred to our immune-oncology gene panel

5) The Discussion further mentions (bottom of page 24):
"Pathway enrichment analysis of the profiled immuno-oncology panel via iDEP and Metascape points towards some dysregulated genes that can modulate the formation of metastatic sites through cytokine signalling".

The text then identifies the cytokine CCL2 as upregulated in cisplatin-resistant tumors and refers to a supplemental figure. Why was this new data only introduced in the Discussion? This is an experimental result. Shouldn't it have been mentioned in Section 3.4?

We decided to keep this information in a Supplemental file because the clinical relevance of this gene did not correlate with development of cisplatin resistance in our cell model. As such, we believe that it would contradict with our gene shortlisting strategy – the expression of the markers should be relevant to patient outcome in the clinical scenario and is not restricted to experimental settings. High expression of CCL2 correlates with better patient survival, while in our models it correlates with high met burden.

6) In the Discussion (page 26), the authors identify that TNFRSF4 "upregulated in the lymph nodes, playing a key role in effector Treg cell development", that "expression was higher in tumour infiltrating T cells than peripheral blood T cells", and "in metastatic melanoma, sentinel lymph nodes had a significantly higher number of OX40+ lymphocytes than healthy lymph nodes expression was higher in tumour infiltrating T cells than peripheral blood T cells".

The authors further indicate that these data "conflict with our observation of decreased TNFRSF4 expression in cisplatin-resistant lymph node metastases in neuroblastoma; however, many of these studies assessed TNFRSF4 expression in lymphocytes within tumour-positive lymph nodes rather than in tumour cells themselves, as we did in our study."

Given this inversed proportion of expression for TNFRSF4 between lymph node lymphocytes and the tumors themselves, is this correlation necessary or advantageous and why would you propose this is occurring

We thank the reviewer for bringing up this interesting question. The main intention of our statement in the discussion was to give an explanation to the apparent conflict between the existing literature about TNFRSF4 pro-oncogenic role and our results. Whether this inverse correlation is necessary and/or sufficient to favour the growth of metastatic cells in lymph nodes can only be determined experimentally. To do so, we should generate murine NB cell lines resistant to cisplatin and repeat the tail-vein injection experiment in fully immune-competent mice. While be believe that this could be an interesting experiment is rather out of scope of the present work. We apologise for not giving more insights in this matter and we added a sentence in the discussion to encourage future work to answer this question. P22

Minor Comments:

1) I am curious why the authors chose SCID Beige mice as a model system for this study. Were nude athymic mice not viable for metastatic modeling?

The frequency of cells with the ability to form metastasis can be very dependent on the mice strain. Thus, along with early studies showing that single menlanoma cells (another neural-crest derived tumours) could form tumours in NOD/SCID IL2gamma mice strain (the most immunosuppressed mice strain) supports that the more immunocompromised is the mice, the easiest is to achieve tumour/metastasis formation (doi: 10.1038/nature07567). Early reports modelling metastasis in neuroblastoma also used the SCID mice strain (10.1002/(SICI)1097-0215(19960729)67:3<379::AID-IJC12>3.0.CO;2-3).

Thus, we decided to use the SCID-beige strain in a compromise of getting sufficient and reproducible metastatic formation rates compared to those that we could possibly get using athymic mice.

2) On page 4 (Section 2.2 of the Materials and Methods), the authors indicate the following:

“Fox Chase SCID Beige mice, which have defective B cells, T cells and NK cells, were injected via tail vein with a suspension of either KellyLuc (cisplatin-sensitive, n=10) or KellyCis83Luc (cisplatin-resistant, n=10) cells.”

Could you indicate how many cells were introduced, the volume, and the characteristics of the “suspension” solution?

We apologize the reviewer for missing this relevant technical information. For the generation of metastasis, 2x10^{6} cells in 150µl of PBS were injected in the lateral tail vein of the mouse. This information is added to P5.

3) Figure 3 (associated with Section 3.2, page 15) appears to have been incorrectly labeled as Figure 1. It is also incorrectly labeled in the text as Figure 1A on page 14 and Figures 1B, C, D, and E on page 16. The actual Figure 1 is identified in Section 3.1 (page 12).

We apologies for this confusion, apparently multiply doc re-sharing adjusted some formatting. We have fixed this issue.

4) On page 21 (Section 3.6), the authors state:
“Owing to the significant inverse correlation shown between MYCN and TNFRSF4 in multiple neuroblastoma datasets (Figure 7A and Supplementary Figure S7)…”
I believe you are referring to Supplementary Figure S6, titled “Correlation between MYCN and TNFRSF4 expression…” which indicates the comparison of the Westermann cohort versus the the Fischer cohort of neuroblastomas.
Supplementary Figure S7 is titled: “Expression of TNFRSF4 and MYCN in a panel of neuroblastoma cell lines.”
As a consequence, Supplementary Figure 6 is not mentioned anywhere in the text and Supplementary Figure S7 needs to be properly introduced.

Many thanks for this comment, we carefully reviewed and fixed this issue.

5) On page 25 (in the Discussion), Supplemental Figure S9 is indicated as associated with an

experiment involving CCL2. There is no Supplemental Figure S9 in the supplementary material. This is Supplemental Figure S8.

Many thanks for this comment, we carefully reviewed and fixed this issue.

6) In the Discussion (page 25), the following statement should probably have a citation to support it:
“The CCL2-CCR2 pair has a long track record of multiple protumorigenic roles, including the promotion of tumour growth and metastasis in many cancer types.”

Many thanks for this comment, we carefully reviewed and added a reference.

Taken together, this manuscript offers a solid set of experiments, but requires a few gaps and corrections be addressed.

Reviewer #2: This manuscript focuses on further characterization of an established cisplastin-resistant neuroblastoma model (KellyCis83), in the context of metastasis, an important consequence and deadly outcome for many patients with high-risk NB. Given that metastatic NB is highly drug resistant, the in vivo RNA sequencing dataset and assessments made regarding TNFRSF4 would provide a valuable resource to the research community.

One concern is the conclusion that TNFRSF4 dysregulation is specific to NB tumor cells. Bulk tumor was dissected, stained and profiled using RNA sequencing. Why is it assumed that leukocytes and/or other stromal cells are not perhaps an additional and/or functional the source of TNFRSF4 in the in vivo model? Please clarify with tissue specific expression profiling from the met biopsies.

For RNA profiling we used a predefined panel of **human** specific immune-oncology genes. Any potential contamination of RNA profiling with RNA from murine tumour microenvironment was eliminated. Additionally, we verified the human TNFRSF4 expression both in Kelly KellyCis83 cell lines alone (RNA and gDNA) and the met biopsies (RNA). We added these clarifications to the relevant sections (Fig S6).

Additionally blast search of Homo sapiens TNF receptor superfamily member 4 (TNFRSF4), RefSeqGene (LRG_1319) on chromosome 1 (NG_046896.1) returned very low similarity with the mouse TNFRSF4/OX40:

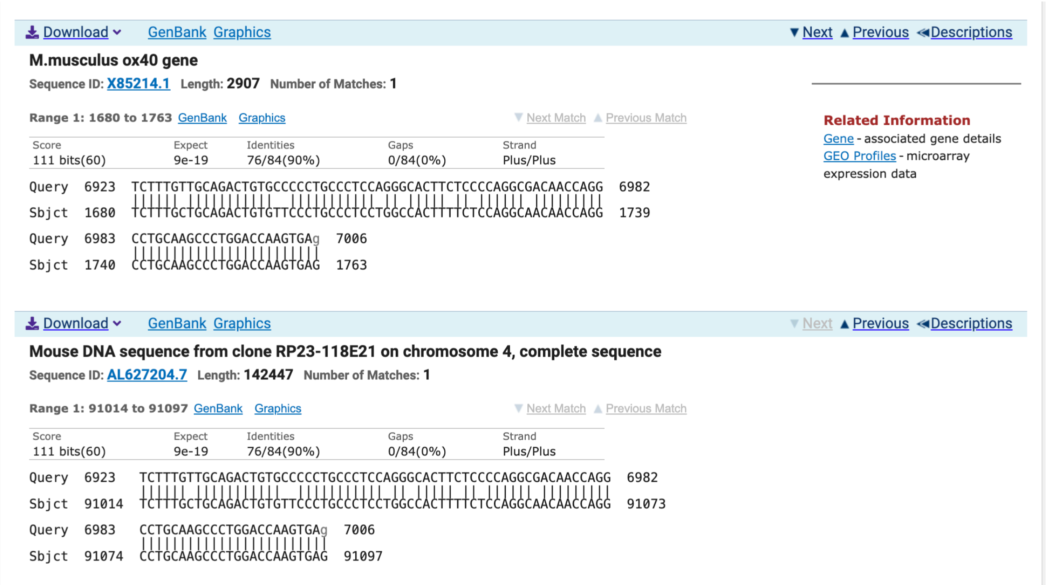
Download GenBank Graphics Next Previous Descriptions

M.musculus ox40 gene

Sequence ID: X85214.1 Length: 2907 Number of Matches: 1

Range 1: 1680 to 1763 GenBank Graphics Next Match Previous Match

Related Information
Gene - associated gene details
GEO Profiles - microarray expression data

Score	Expect	Identities	Gaps	Strand
111 bits(60)	9e-19	76/84(90%)	0/84(0%)	Plus/Plus

```
Query  6923  TCTTTGTTGCAGACTGTGCCCCCTGCCCTCCAGGGCACTTCTCCCCAGGCGACAACCAGG  6982
Sbjct  1680  TCTTTGCTGCAGACTGTGTTCCCTGCCCTCCTGGCCACTTTTCTCCAGGCAACAACCAGG  1739
Query  6983  CCTGCAAGCCCTGGACCAAGTGAg  7006
Sbjct  1740  CCTGCAAGCCCTGGACCAAGTGAG  1763
```

Download GenBank Graphics Next Previous Descriptions

Mouse DNA sequence from clone RP23-118E21 on chromosome 4, complete sequence

Sequence ID: AL627204.7 Length: 142447 Number of Matches: 1

Range 1: 91014 to 91097 GenBank Graphics Next Match Previous Match

Score	Expect	Identities	Gaps	Strand
111 bits(60)	9e-19	76/84(90%)	0/84(0%)	Plus/Plus

```
Query  6923   TCTTTGTTGCAGACTGTGCCCCCTGCCCTCCAGGGCACTTCTCCCCAGGCGACAACCAGG  6982
Sbjct  91014  TCTTTGCTGCAGACTGTGTTCCCTGCCCTCCTGGCCACTTTTCTCCAGGCAACAACCAGG  91073
Query  6983   CCTGCAAGCCCTGGACCAAGTGAg  7006
Sbjct  91074  CCTGCAAGCCCTGGACCAAGTGAG  91097
```

This approach together with the Fox Chase SCID Beige mice immune background, which have defective B-cells, T-cells and NK cells but functioning macrophages it was possible examine basal expression of immune-oncology related genes in neuroblastoma cells with minimal interaction with the immune system. We excluded a potential cross talk with the other murine stromal cells because of the low similarity between human and mouse TNFRSF4 genes.

Related to the previous comment regarding the potential for TNFRSF4 expression in stromal tissues, scRNAseq data from NB patients (Wienke et al. Cancer Cell 2024) show TNFRSF4 expression in NB-associated Tregs and potentially an immune suppressive activity by this cell type. This recently published data might lead one to guess that increased stromal TNFRSF4 expression associates with worsened outcomes, which contradicts the data shown in Fig 4. It may be a consideration to discuss this published finding as it relates to the current manuscript, KellyCis83 and TNFRSF4.

We appreciate this comment and reference to Wienke et al. Cancer Cell 2024. We carefully reviewed this suggestion and think it does not fit the current story. The clarifications above should have shed light on the altered TNFRSF4 expression. However, we updated our discussion with this reference as appropriate.

Even though characterization of these Kelly cell lines was previously published, it would be beneficial to comment in section 3.1 or elsewhere on differences between Kelly and KellyCis83Luc with respect to growth dynamics and genomic changes (chromosomal gains, losses, rearrangements). For example, are any changes consistent with chromosomal loss discussed in patients on pg. 18?

We thank the reviewer for this comment. We carefully reviewed and added further information. P 4

Is there an explanation for why the RT-PCR data (pg. 15, Fig. 1 C and E) are inconsistent with

the RNA sequencing. Is it that these genes differentially expressed in stromal cells? This could be further explored on tumour section and/or clarified in the manuscript.

A bench mark study has been performed to validate RNA-sequencing analysis workflows using RT-qPCR. The study compared RNA-sequencing data, processed using five workflows with expression data generated by wet-lab validated qPCR assays for 18 080 protein-coding genes. The comparison demonstrated that about 85% of the genes showed consistent results between RNA-sequencing and qPCR data. Genes that were typically lower expressed, smaller and had fewer exons demonstrated poor consistency. The authors concluded that careful validation is warranted when evaluating RNA-seq based expression profiles. [doi: 10.1038/s41598-017-01617-3]
It is likely that SOCS3 met the latter criteria. SOCS3 has only 2 Exons, of which only one is coding Transcript: ENST00000330871.3 SOCS3-201.
TNFRSF1B has several transcripts which vary a lot ENST00000536782.2 Exons: 5, Coding exons: 5,
ENST00000489921.1 Exons: 3, Coding exons: 0
ENST00000376259.7 Exons: 10, Coding exons: 10
ENST00000492361.1 Exons: 9, Coding exons: 0
These variations may explain the lack of correlation between RNAseq data and RT-qPCR validation.

In the discussion, it is mentioned that the tumor size of KellyCis83 mets are increased. It would be interesting to present this data in Fig 1 to compliment met number.

We apologize for the confusion that our statement in the discussion section may have generated. As it mentioned in the methods and the results section, we scored the number of metastases but we did not measure their size or weight them. We have modified the discussion paragraph and now reads like this:

“One of the most intriguing observations is an almost twofold increase in metastatic aggressiveness of cisplatin-resistant KellyCis83Luc mets compared to the cisplatin-sensitive control group. While an increase in the number of detectable metastases of KellyCis83 could be explained by increased doubling time(13) and alterations in the cell cycle(25), their invasion potential *in vivo* does not correlate with the same *in vitro*.” P20

It does not look like the stiffness data presented in Fig.1F is significantly different in Kelly versus KellyCis83?. While this may be a possible explanation for the observed phenotypic changes, without significant changes, this reviewer does not fully understand the value of presenting and discussing this data in the manuscript. A p-value in Fig.1F may help clarify this point.

We carefully reviewed and added further information to Fig 1: a p-value and a number of mets examined. KellyLuc n=2 and KellyCis83Luc n=5. P11

Minor comments:
-"Figure 1: Functional enrichment analysis..." (pg.15) figure legend and associated references in the text appear mislabelled and based on the presented sequence should be

Figure 3 vs Figure 1? We apologies for this confusion, apparently multiply doc re-sharing adjusted some formatting. We revised it.
-A legend for "dot sizes correspond to adjusted p values" in Fig1 (maybe Fig.3, pg 15) could be helpful - We carefully reviewed and added further information.
-Reference the source of "large cohort of 498 neuroblastomas" on page 16, line 2. We added the reference GEO to this dataset in Materials and Methods.
-Raw data from the RNA sequencing do not appear to be deposited in a publicly available database. Sharing of these data would facilitate their use in other studies.

We have deposited this RNA sequencing data “Neuroblastoma KellyLus-Kelly83Luc xenografts, the HTG EdgeSeq immuno-oncology panel” at R2: Genomics Analysis and Visualization Platform and made it publicily available. P24

6. PLOS authors have the option to publish the peer review history of their article (what does this mean?). If published, this will include your full peer review and any attached files.

If you choose “no”, your identity will remain anonymous but your review may still be made public.

Do you want your identity to be public for this peer review? For information about this choice, including consent withdrawal, please see our Privacy Policy.

Reviewer #1: No

Reviewer #2: No
